# Coronavirus Disease 2019 (COVID-19) in Cancer Patients

**DOI:** 10.7759/cureus.7835

**Published:** 2020-04-26

**Authors:** Ivan Cancarevic, Praveena Tathineni, Bilal Haider Malik

**Affiliations:** 1 Internal Medicine, California Institute of Behavioral Neurosciences and Psychology, Fairfield, USA; 2 Internal Medicine, University of Illinois at Chicago/Advocate Christ Medical Center, Chicago, USA

**Keywords:** sars-cov-2, wuhan coronavirus, novel coronavirus, coronavirus disease, cancer patients, coronavirus pandemic, severe acute respiratory syndrome coronavirus 2, 2019 novel coronavirus, lung cancer, cancer

## Abstract

The Coronavirus disease 2019 (COVID-19), caused by severe acute respiratory syndrome coronavirus 2 (SARS-CoV-2), has become the most talked-about clinical entity in early 2020. As an infection that spreads easily and has a significant mortality rate, it has caused global panic rarely seen before. Many of the measures taken by governments worldwide will have long-lasting impacts on the wellbeing of the population at large. It has been widely reported that the most vulnerable patients have been most negatively affected by SARS-CoV-2 (COVID-19). In this study, we have tried to search the currently available data on the outcomes of infected cancer patients. Most of the data points to the very challenging nature of treating such patients. Their overall outcomes seem to be worse than in the general population, and it may be difficult to differentiate which potential complications are a result of the primary oncologic disease versus the infection. Management presents its own set of challenges, including but not limited to, deciding whether postponing cancer treatment until the infection resolves is going to benefit the patient and how to organize all aspects of patient care when social contact is as limited as it is for patients newly diagnosed with COVID-19. We believe that as more data becomes available, it is going to be necessary to publish detailed guidelines on how to approach this unique clinical challenge.

## Introduction and background

The Coronavirus disease 2019 (COVID-19), caused by severe acute respiratory syndrome coronavirus 2 (SARS-CoV-2), was first described in Wuhan, China, in December 2019 [1]. After severe acute respiratory syndrome coronavirus (SARS-CoV) and Middle East respiratory syndrome coronavirus (MERS-CoV), SARS-CoV-2 is the third highly pathogenic and transmissible coronavirus [2]. Since December, the virus has spread globally and has led to the implementation of some of the most serious policies around the world. On 30 January 2020, it was declared a public health emergency by the World Health Organization (WHO) [3]. Since then, we have seen a number of consequences that have impacted people's lives. On 11 March, the United States restricted the entry of European citizens into the country [4]. Since then, multiple countries have suspended all but essential travel and strongly advised their own citizens against overseas travel [5]. The economic impact has also been huge. Financial markets have been severely impacted, putting people's financial wellness at risk [6]. The incubation period is not notably longer than for other viruses in the same family [1]. However, the fact that people have been required to stay in isolation for 14 days has had a severe psychological impact on many affected individuals [7].

The most common clinical presentation is that of a mild upper respiratory tract infection, although a number of patients develop progressive respiratory failure [8]. Cough, fever, and fatigue are the most frequently reported presenting symptoms, evident in the vast majority of cases, followed by expectoration, myalgia, and gastrointestinal symptoms [9-11]. The most common co-morbidities of patients diagnosed with COVID-19 were hypertension and diabetes mellitus, while respiratory co-morbidities were comparably rare [9-11]. Chest X-Rays showed evidence of bilateral ground-glass or patchy opacities in almost 90% of patients, while leukopenia and, to a lesser extent, eosinopenia were frequently reported laboratory findings [9-11]. Severe disease was associated with elevated levels of C-reactive protein, D-dimer, and procalcitonin [11]. The risk of intensive care unit (ICU) admission and death was significantly higher in older individuals and those with co-morbidities and the development of end-organ damage (acute respiratory distress syndrome, acute kidney injury, cardiac injury) [9]. Many treatment regimens have been attempted, without a clear strategy being developed at the time of this writing. Empiric antivirals, antibiotics, corticosteroids have been frequently used [11].

The purpose of this article is to analyze the impact of COVID-19 infection on patients with malignant co-morbidities. Considering the increased severity of the disease in those patients, their social, psychological and physical limitations and the number of such patients in the society, we strongly believe that it is essential to get a clearer understanding of the unique challenges in protecting some of the most vulnerable members of the society during this outbreak.

## Review

Reports of cancer patients infected with SARS-CoV-2

Cancer patients have weaker immune systems compared to the general population, both due to the disease itself as well as the treatment. As such, morbidity and mortality of any serious infections would be expected to be high among cancer patients. Liang et al. found that the prevalence of cancer among patients infected with SARS-CoV-2 (COVID-19) was higher than in the general population [12]. One percent of the COVID-19 cases had a history of cancer when compared to 0.29% of the Chinese population [12]. Lung was the most common primary site with 5/18 patients having lung cancer [12]. Colorectal (4/18), breast (3/18), and bladder (2/18) cancer were reported more than once, while lymphoma, papillary thyroid cancer renal cell carcinoma, and adrenal carcinoma were reported once [12]. Compared to other patients with confirmed COVID-19 infections, cancer patients were, on average older, more likely to be smokers, and had more severe baseline CT findings while having similar baseline X-Ray findings, symptoms, and other co-morbidities [12]. They also reported that the outcomes (including the likelihood of ICU admission) of such patients were, on average worse when compared to those without a malignant co-morbidity (39% versus 8%) [12]. Those who underwent surgical resections or chemotherapy within the past month were also found to be more likely to die or require ICU admission (3 out of 4 versus 6 out of 14) [12]. Xia et al., however, raised a few concerns about the findings in the study published by Liang et al. [13]. They believe that the sample size of only 18 cancer patients infected with SARS-CoV-2 is too small and also that Liang et al. failed to account for possible confounders, including smoking/chronic obstructive pulmonary disease (COPD) history which is an independent risk factor for worse outcomes in COVID-19 infections [13-14]. Diagnostically, COVID-19 infections complicate the diagnosis of lung infiltrates in cancer patients [15].

Overall, it is clear that the recent outbreak significantly affects cancer patients. The association between malignant co-morbidity and mortality from COVID-19 is less clear. Smoking status and other systemic, especially pulmonary co-morbidities, potentially skew the mortality rate. Also, the widespread presence of another condition that presents with lung infiltrates adds to the difficulty of accurately diagnosing and treating those patients. We would encourage clinicians to keep reporting any new cases so that a larger systematic review can be done, and possible confounders, especially smoking and other lung diseases, can be addressed.

The impact of social isolation on cancer patients

Cancer patients are known to be prone to developing psychiatric disease, including anxiety and depression. As such, mental health care is an important aspect of treating patients suffering from malignant disorders. Considering the importance of social contacts for cancer patients, the quarantine requirements for patients suspected of contracting SARS-CoV-2 could further complicate their situation. Kotronoulas et al. found that emotional support and reassurance were the most prominent needs of colorectal cancer patients regardless of the disease stage of the treatment phase [16]. Faller et al. reported that psycho-oncologic interventions were associated with effects on emotional distress and quality of life with longer interventions yielding longer-lasting results [17]. The study was limited by small-sample bias and low-quality reporting in some of the studies analyzed [17]. Moore et al. discovered that the impact of social isolation on cancer survival was significant (HR=1.06 p=0.04), even when accounted for the fact that patients receiving no therapy were more likely to be socially isolated [18].

Multiple studies suggest that the outcomes of cancer patients are dependent on their social infrastructure [16-18]. Nowadays, psychological support is becoming an integral part of comprehensive cancer management [19]. With the number of people in isolation rising due to the SARS-CoV-2 outbreak, it is reasonable to assume that the issue of social isolation is going to become more prominent, which is especially worrisome for cancer patients. Recently, there have been news reports on quarantined patients in Spain being found 'dead and abandoned,' which provides valuable insight into the scale of the problem [20]. It seems evident that hospitals and practitioners should work on developing systems to help cancer patients maintain as much social contact as possible throughout the outbreak. Psychological interventions through phone and video calls may prove to be an essential part of the management of such patients.

Management

Managing cancer patients during the COVID-19 outbreak presents multiple unique challenges. The amount of time cancer patients spend at hospitals, the hospital schedule changes, and the necessity of administering chemotherapy and radiotherapy, which weaken the immune system, all increase the difficulty of properly treating those patients. As Liang et al. pointed out, cancer history, surgery, and chemotherapy all significantly contributed to adverse outcomes [12]. A possible problem with the study is that the number of patients was low [12]. Interestingly, lung cancer did not appear to be more hazardous than other forms of cancer [12]. Yu et al. stressed the importance of adequate protection for health workers during abdominal surgeries [21]. Also, they believed that the laparoscopic approach was preferable over the traditional approach [21]. Once a cancer patient is diagnosed with COVID-19, it is debatable whether cancer treatment should be continued while the patient is recovering from the infection [22-23]. Zhang et al. reported a case of a lung cancer patient whose osimertinib treatment was continued throughout the infection due to his stable clinical condition, and the patient made a complete recovery and was discharged [22]. Yang et al. point out that generally, anti-COVID-19 therapy should be either combined with cancer therapy or administered prior to it [23]. They recommend that integrative cancer therapies that involve close contact with patients are forbidden or only permitted in rare circumstances [23].

Despite the relative scarcity of the available data due to the novelty of the SARS-CoV-2, it remains clear that the majority of experts recommend focusing on infection management over cancer management. If new studies show that there is a significant association between chemotherapy- or radiotherapy-induced immunosuppression and adverse outcomes of COVID-19 patients, a short delay in administering them would likely be beneficial in order to improve the odds of surviving the acute infection. However, it should be noted that most of the currently available articles used the data from Liang et al. and as Xia et al. pointed out, there are multiple issues with that study [12-13]. We would strongly encourage clinicians to keep reporting any cases of cancer patients infected with SARS-CoV-2, their management, and the outcome in order to further our understanding of this complex issue.

## Conclusions

The outbreak of COVID-19 has caused global panic rarely seen before and reaching management decisions has been difficult due to the scarcity of available data on the disease and the lack of clearly known risk factors for adverse outcomes. The currently available evidence seems to suggest that there is a correlation between cancer and COVID-19 as cancer prevalence among the infected patients seems higher than in the general population, with lung being the most frequent site. Since most of the affected individuals have few symptoms of the infection, it poses a diagnostic challenge to determine the exact etiology of lung infiltrates in cancer patients. Smoking and other chronic conditions could be confounders that need to be addressed. Lack of social interactions is another problem facing cancer patients at the time of the SARS-CoV-2 outbreak as social isolation is the most important infection prevention measure. Finally, a challenge worth pointing out is the issue of delaying cancer treatment, especially in patients with mild cases of COVID-19. The currently available evidence suggests that oncologic therapies may be acceptable in those with mild infections. That being said, it is still impossible to tell whether patients would benefit from it, and the additional risk to healthcare workers could make such practice more hazardous than delaying cancer treatment until the infection resolves. We believe that it is essential that clinicians keep reporting cases of COVID-19 in patients with malignant co-morbidities. More detailed guidelines will be necessary to determine the priorities in treating those patients.
